# Non-invasive Prediction of Meningioma Tumor Grade by Quantification of Shape-based Radiomics Features and Surface Regularity

**DOI:** 10.1007/s00062-025-01560-1

**Published:** 2025-09-17

**Authors:** Martin Diebold, Lucas Becker, Theo Demerath, Marco Reisert, Daniel Erny, Andreas Braun, Till-Karsten Hauser, Jürgen Grauvogel, Marc Hohenhaus, Horst Urbach, Alexander Rau, Urs Würtemberger

**Affiliations:** 1 Institute of Neuropathology, Medical Center—University of Freiburg, Faculty of Medicine, University of Freiburg, 79106 Freiburg, Germany; 2 Departments of Neurology and medical Oncology, University Hospital Basel, Petersgraben 4, 4031 Basel, Switzerland; 3 IMM-PACT Clinician Scientist Program, Faculty of Medicine, University of Freiburg, 79106 Freiburg, Germany; 4 Department of Neuroradiology, Medical Center—University of Freiburg, Faculty of Medicine, University of Freiburg, Breisacher Straße 64, 79106 Freiburg, Germany; 5 Department of Stereotactic and Functional Neurosurgery, Medical Center—University of Freiburg, Faculty of Medicine, University of Freiburg, 79106 Freiburg, Germany; 6 Department of Medical Physics, Medical Center—University of Freiburg, Faculty of Medicine, University of Freiburg, 79106 Freiburg, Germany; 7 Department of Diagnostic and Interventional Neuroradiology, University Hospital Tuebingen, 72076 Tuebingen, Germany; 8 Department of Neurosurgery, Medical Center-University of Freiburg, University of Freiburg, Freiburg, Germany

**Keywords:** MRI, Tumor shape, Non-invasive biomarker, Meningioma grading, Radiomics

## Abstract

**Purpose:**

Prognosis and therapeutic approaches to meningiomas are determined by their proliferative activity and tumor grade. The early identification of aggressive phenotypes is hence essential to improving outcomes. Here, we investigated the potential of shape-based radiomics features and surface regularity (SR) as non-invasive biomarkers of atypical (grade 2) and anaplastic (grade 3) meningiomas.

**Methods:**

In this retrospective single-center study, we assessed individuals with treatment-naive meningiomas in a comprehensive analysis of MR imaging with histopathological grading and quantification of mitotic activity. Radiomics (sphericity, elongation, flatness) and SR measures were calculated on segmented contrast-enhancing tumor components and tested for association with WHO grade (*n* = 62 grade 1, *n* = 71 grade 2, and *n* = 19 grade 3) and proliferation.

**Results:**

All tumor grades were significantly different in characteristics of sphericity and SR with decreasing values at higher tumor grades as an expression of a more irregular shape. Additionally, radiomics features of tumor elongation and flatness discriminated grade 3 cases from grade 1 (*p* = 0.0008 and *p* = 0.0003) and grade 2 meningiomas (*p* = 0.0008 and *p* = 0.0013). Mitotic activity exhibited a significant negative correlation with the four imaging markers assessed (*p* < 0.003).

**Conclusion:**

Shape-based radiomics features of sphericity and SR can serve as preoperative grading biomarkers of meningiomas in routine contrast-enhanced T1w MRI.

## Introduction

Meningiomas constitute the most frequent intracranial neoplasm and are categorized into three tumor grades based on histopathological features and molecular markers: benign grade 1 (80.3%), atypical grade 2 (17.9%) and rare anaplastic grade 3 (1.6%) [1]. Currently, the primary criteria for evaluating the risk of recurrence are WHO tumor grade and the extent of resection [2]. Benign meningiomas have a more favorable course and good local control once complete tumor resection has been achieved. In contrast, atypical and anaplastic meningiomas have a higher tendency to recur and poorer survival rates [3]. Therefore, adjuvant therapy should be considered in grades 2 and 3, as well as complementary or standalone radiosurgery or radiotherapy [4]. The mitotic rate constitutes a fundamental component of the WHO surface reclassification system for grading. Though it exhibits variability within grades, it possesses independent prognostic significance [5, 6] and may serve as an indicator of malignant transformation [7].

The quantification of the mitotic rate requires biopsy material for processing. However, non-invasive prediction of tumor grade is feasible via imaging as shown by a study on 500 patients with intracranial meningioma. Here, a correlation between peritumoral edema volume, heterogeneous contrast enhancement pattern, and an irregular tumor shape in high-grade meningiomas was noted [8]. However, both the tumor shape (regular or irregular) and contrast enhancement (homogeneous vs. heterogeneous) were only evaluated in binary terms, and no differentiation was made between atypical and anaplastic meningiomas.

Tumor shape is objectively assessable through so-called surface regularity (SR). SR allows to quantify the extent to which a segmented tumor volume deviates from the surface of an ideal, spherical shape of the same volume [9]. SR was demonstrated to be an independent predictor of survival in patients with glioblastoma [9, 10]. Methodologically related to SR, a study found a significantly lower tumor surface factor in a combined group of grade 2 and 3 meningiomas compared with grade 1 meningiomas. Further subdifferentiation between grade 2 and 3 was not achieved due to the small number of anaplastic meningiomas [11].

Radiomic approaches have been employed to quantify shape features within tumor segmentations. Here, a meta-analysis demonstrated that radiomics-based preoperative grading of meningiomas using various machine learning algorithms appears to be feasible [12]. However, a common feature of the investigated studies was the dichotomous classification of WHO grade 1 versus WHO grade 2 and 3. For example, lower sphericity appears to be associated with parameters of aggressive meningioma biology such as somatic mutation burden and DNA methylation status and can distinguish between low-grade and high-grade meningioma [13]. Higher-grade meningiomas exhibit more irregular tumour growth and a higher propensity for brain invasion [14], thus being more likely to induce changes in shape.

In recent research, it was demonstrated that a prediction of the integrated molecular and morphologic risk score (IRS) is feasible in higher-grade meningiomas (grade 2 and 3) using shape-based radiomics feature sphericity. This approach enables enhanced diagnostic accuracy in patients exhibiting an elevated risk of progression, providing a non-invasive method for risk stratification. However, benign meningiomas were not included in the study mentioned here [15].

Here, we investigated the potential of shape-based radiomics features and SR as non-invasive biomarkers for predicting tumor grade and distinguishing between all three tumor grades.

## Materials and Methods

### Patients and Data Collection

**Fig. 1:**
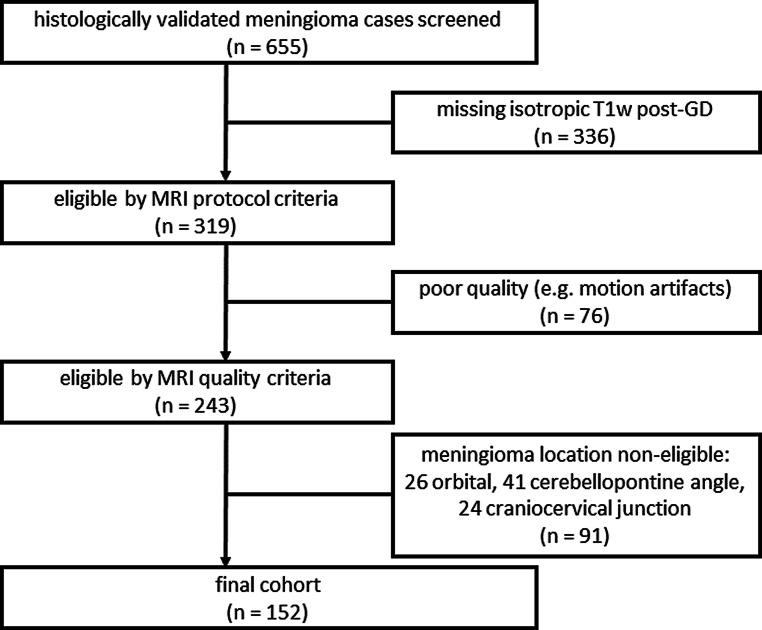
Flow-chart of screening procedure and enrolled cases

In this single-center study at a tertiary referral center, we retrospectively screened our MRI database for patients with histopathologically confirmed treatment-naive intracranial meningiomas (WHO grade 1, 2, and 3) and available presurgical isotropic T1w post-GD MRI sequences. We carefully excluded patients with limited image quality that could compromise the quality of the segmentations, and tumor locations involving the orbita, cerebellopontine angle and craniocervical junction (Fig. 1). The study was conducted in accordance with the 1964 Helsinki Declaration and its later amendments and approved by the local ethics committee (EK:400/20) and informed written consent was waived.

### *Image Postprocessing*

**Fig. 2:**
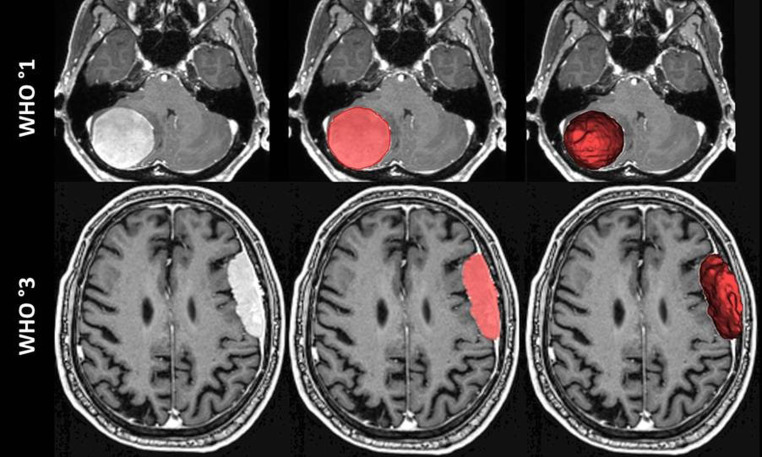
Non-invasive analysis of brain tumor to aid in accurate tumor grading. Axial reformat of a T1w post-Gd MRI of a right cerebellar benign meningioma (top row) and a left frontal anaplastic meningioma (bottom row) with unprocessed data on the left, tumor segmentation of the respective slice in the middle and superimposed 3D tumor segmentation on the right. The grade 1 meningioma demonstrates a regular, rounded shape with higher SR and sphericity. In contrast, the grade 3 meningioma exhibits an irregular, elongated and less round shape, accompanied by lower SR and sphericity

Segmentations were carried out on preoperative T1w sequences (isotropic resolution 1 mm^3^) after intravenous administration of a single dose of gadubutrol (0.10 mmol/kg) using 1.5 and 3T MRI scanners. In order to enable precise tumor delineation, a cross-check was always performed with the available, non-contrast-enhanced 2D or 3D T1-weighted sequences. Image data was imported to a local instance of the NORA post-processing platform (www.nora-imaging.org). Each patient was individually assessed for sufficient image quality and those with artifacts (e.g. due to movement) were excluded. Contrast-enhancing tumor components were manually segmented by a neuroradiologist in training and a board-certified neuroradiologist (with 6 and 8 years of experience in clinical neuroimaging) in consensus based on triplanar reformats (an example is provided in Fig. 2). From these segmentations, we extracted shape-based radiomic features using PyRadiomics 3.1.0 (https://pyradiomics.readthedocs.io/en/latest/features.html) [16], information about data and code is available on GitHub repository (https://github.com/AIM-Harvard/pyradiomics). While there are 16 (3D) shape-based radiomics features and 10 (2D) shape-based radiomics features, we have deliberately only analyzed the three 3D shape-based radiomics features sphericity, flatness and elongation for reasons of comprehensibility, as these represent relative values that are independent of tumor size. Additionally, we calculated a measure of SR using the following formula according to Pérez-Beteta et al.[9]:

$$S_{R}=6\sqrt{\pi }\frac{TV}{\sqrt{\left(TS\right)^{3}}}$$

in which TV is the total volume (in cm^3^) and TS is the total surface area (in cm^2^). SR is a dimensionless ratio that compares the segmented tumor volume to the volume of a sphere of equivalent surface area, which ranges from 0 (indicating highly irregular, fractal-like tumor surfaces) to 1 (indicating spherical tumors).

### *Histopathology*

The initial histopathological workup was performed according to standard operating procedures at the institute of neuropathology of the tertiary referall center following WHO criteria for classification of CNS tumors. All samples underwent histopathologic classification, including determination of the mitotic index and assessment of infiltration of adjacent tissue (specifically infiltration of the central nervous system) since mitotic activity, infiltrative growth and micro-morphology largely define the grading of menigiomas. In short, sections of 4 μm thickness were processed and stained according to hematoxylin/eosin protocols and subjected to eventual genetic examination for mutations associated with grade 3 meningiomas and/or immunohistochemical analyses to confirm CNS infiltration (by glial fibrillary acidic protein or synaptophysin) or elevated proliferation rate (by protein KI-67) where indicated. At the time of diagnosis, grading was conducted according to the most recent WHO CNS tumor classifications [17].

### *Statistical Analysis*

Statistical analysis was performed using the graphing and statistics software application GraphPad Prism (Boston, MA; version 10.3.0). Linear regression models were used to assess the association between the mitotic rate and the radiomic features sphericity, flatness, elongation, and SR, respectively. Coefficients of determination (R^2^) and *p*-values were used to evaluate strength and significance of potential associations. The same radiomic features were assessed by WHO grade of the tumor sample using two-tailed t‑tests. To evaluate the diagnostic potential of each shape feature and SR in differentiating the WHO grades, receiver operating characteristics (ROC) were plotted and compared by the area under the ROC curve (AUROC). Formal significance was considered at a level of 0.05.

## Results

A total of 152 meningiomas were assessed in this cohort (62.4% female) and a mean age of 60.0 years (range 12–91 years). Of those, 62 tumors were diagnosed as grade 1 by neuropathological work-up as well as 71 tumors of grade 2 and 19 tumors of grade 3, respectively (Table 1). No significant group differences between WHO grades were noted for age or sex.

**Table 1:** Cohort characteristics stratified by CNS WHO grade.

CNS WHO grade	Grade 1	Grade 2	Grade 3
Individuals	62	71	19
Mean age (ränge)	58 (32–82)	63(12–86)	67 (34–91)
Sex (female: male)	44: 18	40: 31	11: 8
*Location (n (%))*			
Convexity	24(39)	31 (44)	5(26)
Falcine/parafalcine	16 (26)	20(28)	10(53)
Skull base	15(24)	16 (23)	2(11)
Tentorium/fossa posterior	6(19)	2(3)	2(11)
Intraventricular/other	1(2)	2(3)	0
*Mitotic rate*			
Mean	0.83	2.73	24.84

**Fig. 3:**
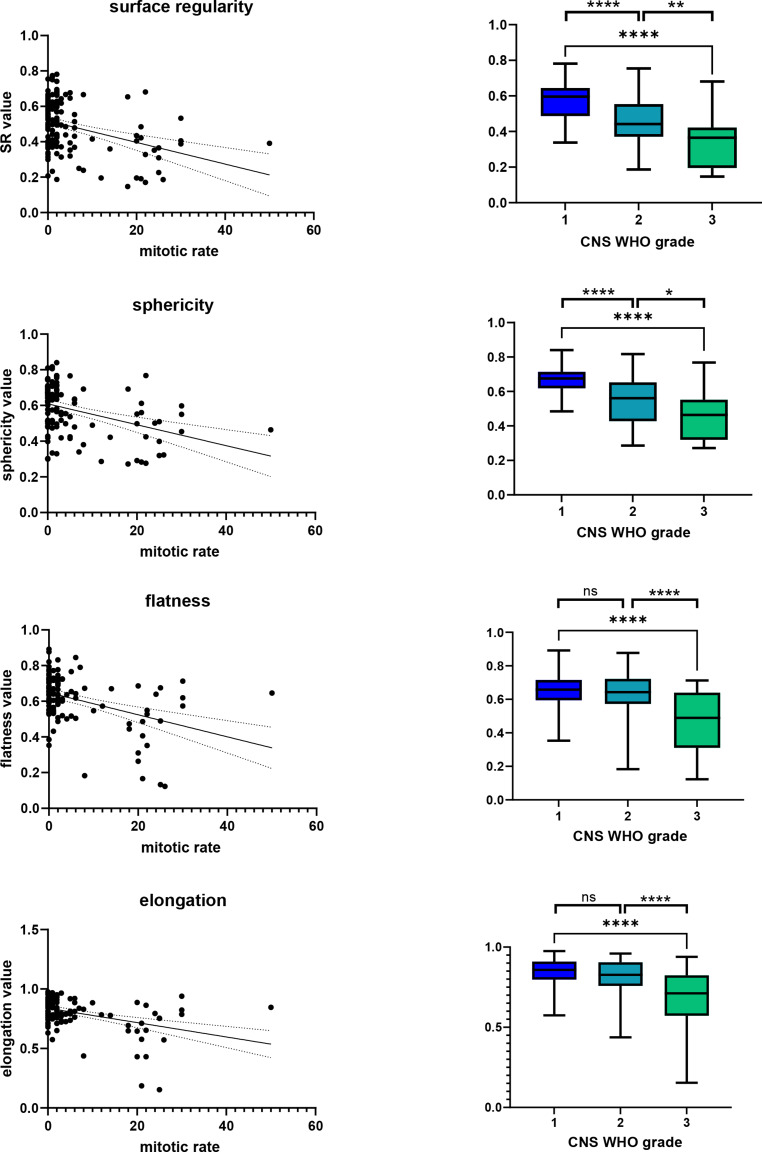
Surface properties and radiomically assessed shape features correlate with proliferation rate and show robust associations with WHO tumor grade. SR and shape features sphericity, flatness and elongation, inversely correlate with the mitotic rate of the histopathologically assessed tumor sample (left column) and the tumor grade as defined by WHO CNS tumor classification (right column)

We initially assessed a potential association of SR with the proliferative activity of meningiomas. In a linear regression model, we identified a significant negative association of mitotic rate and SR (R^2^ = 0.056, *p* = 0.0063). In line with this observation, sphericity (R^2^ = 0.08484, *p* = 0.0017), flatness (R^2^ = 0.078, *p* = 0.0025) and elongation (R^2^ = 0.077, *p* = 0.0029) weakly related to the histologic measure of mitotic figures per high power field (Fig. 3 left panel).

Since elevated proliferation constitutes a hallmark of higher grade meningiomas, we next tested whether the four previously identified correlations translate into associations with CNS WHO tumor grades on a group level. One-way ANOVA tests for each of the four parameters indicated significant differences between the three CNS WHO grades (for SR: F = 24.9 and *p* < 0.0001, for sphericity: F = 21.7 and *p* < 0.0001, for flatness: F = 15.7 and *p* < 0.0001, for elongation: F = 17.1 and *p* < 0.0001). In direct comparison of each two tumor grades for SR, comparison of grades 1 vs. 3 (*p* < 0.0001), 1 vs. 2 (*p* = 0.015) and 2 vs. 3 (*p* = 0.0024) showed a robust separation with mean values of 0.59 (grade 1), 0.55 (grade 2) and 0.45 (grade 3). Similarly, sphericity allowed separation of CNS WHO grades 1 vs. 3 (*p* < 0.0001), 1 vs. 2 (*p* = 0.001) and 2 vs. 3 (*p* = 0.008) with mean values of 0.69 (grade 1), 0.63 (grade 2) and 0.55 (grade 3). Flatness and elongation shape features both did not allow significant discrimination of tumor grades 1 vs. 2 (*p* = 0.37 and *p* = 0.16 for elongation, respectively), but clearly separated grades 1 vs. 3 (*p* = 0.0003 for flatness and *p* = 0.0008 for elongation) and grades 2 vs. 3 (*p* = 0.0013 for flatness and *p* = 0.0008 for elongation). Mean absolute measures were 0.66 (grade 1), 0.65 (grade 2) and 0.52 (grade 3) for flatness, and 0.85 (grade 1), 0.84 (grade 2) and 0.73 (grade 3) for elongation (Fig. 3 right column).

**Fig. 4:**
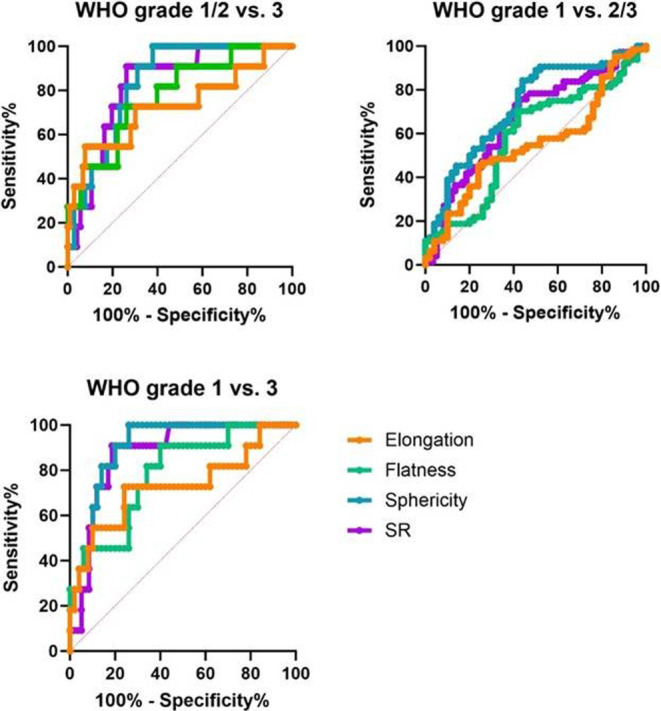
Morphometric parameters show differential accuracy profiles in discriminating CNS WHO grades. Receiver-operator-curves depicting sensitivity and specificity of surface features SR (purple) and radiomics features elongation (orange), flatness (green) and sphericity (blue) for the discrimination of tumor grades

Finally, we employed ROC to test the accuracy of discrimination between tumor grades based for each parameter alone. SR and sphericity showed highest areas under the ROC curve (AUROC) for discrimination of WHO grade 3 meningiomas from benign WHO grade 1 meningioma cases (AUROC (95% CI, *p*-value)): SR 0.88 (0.79–0.98, *p* = 0.0001), sphericity 0.91 (0.79–0.99, *p* < 0.0001), flatness 0.79 (0.69–0.93, *p* < 0.0001), elongation 0.73 (0.68–0.93, *p* = 0.0001). Similarly, SR and sphericity performed well in discriminating grade 1 and grade 2 meningiomas from anaplastic grade 3 meningiomas (SR 0.79 (0.69–0.91, *p* < 0.0001), sphericity 0.79 (0.67–0.90, *p* < 0.0001)), whereas flatness and elongation performed good to modest (flatness 0.78 (0.66–0.90, *p* < 0.0001), elongation 0.77 (0.65–0.90, *p* < 0.0001)). Interestingly, both SR and sphericity performed modestly in discriminating grade 1 from “high-grade” 2 and 3 meningiomas (SR 0.75 (0.67–0.83, *p* < 0.0001), sphericity 0.79 (0.71–0.87, *p* < 0.0001)) while flatness and elongation did not suggest suitability for discrimination (flatness 0.62 (0.52–0.72, *p* = 0.03), elongation 0.64 (0.54–0.74, *p* = 0.01)) (all ROC curves Fig. 4). Potential cut-offs for identification of grade 1 meningiomas are an SR-value > 0.5 (sensitivity 67%, specificity 70%) and a sphericity-value > 0.6 (sensitivity 66%, specificity 78%). For identification of grade 3 meningiomas cut-offs of an SR-value < 0.45 (sensitivity 85%, specificity 67%), a sphericity-value < 0.50 (sensitivity 64%, specificity 77%), a flatness-value < 0.6 (sensitivity 68%, specificity 71%) or an elongation-value < 0.8 (sensitivity 74%, specificity 66%) were quantified.

## Discussion

In a well-characterized cohort, we found that shape-based radiomics features and SR differed between WHO tumor grades in meningiomas. In addition, there was a negative correlation between shape-based features and SR with the mitotic rate in meningiomas.

Several studies investigated associations between meningioma tumor grades and imaging features, including the subjectively assessed more irregular tumor shape as a semi-quantitative parameter [8, 21] for higher tumor grade. However, within the high-grade meningiomas, atypical and anaplastic meningiomas were not yet separately analyzed. Here, we achieved delineation of these entities primarily due to the sufficient number of anaplastic meningiomas in our cohort. This non-invasive approach may prove beneficial as it streamlines patient pathways. The planned extent of the resection and possible adjuvant therapies could be estimated with a higher degree of confidence. While resection is usually the treatment of choice for atypical meningiomas, no randomized studies are available on the use of fractionated radiation. In the case of anaplastic meningiomas, resection should be followed by fractionated radiation [4]. In cases of incidentally diagnosed meningiomas in asymptomatic patients, follow-up monitoring is the treatment of choice, even though this does not allow for histological confirmation [4, 19, 20]. Non-invasive tumor grading is becoming increasingly important in this context. Similarly, if resection is contraindicated, radiosurgery may be considered [20] and, in the absence of histology, non-invasive tumor grading using SR and sphericity may also be relevant, particularly to help characterize clearly malignant anaplastic meningiomas.

In light of their widespread availability, we conducted a focused analysis of only 3D T1w post-GD sequences, and examined shape-based radiomic features in addition to SR exclusively. These features have the great advantage that they are the most independent of the contrast agent dynamics, circulatory parameters or the MRI scanner used, and thus enable good generalizability. Additionally, they describe easily comprehensible characteristics of the tumor shape, which distinguishes them from other radiomic feature classes [21, 22]. These other classes are dependent on gray values, such as the first order or histogram features, as well as the texture features. As a result, they are more difficult to understand as already visually recognizable shape differences [23].

All three tumor grades showed significant differences in sphericity and SR with decreasing values at higher tumor grades as an expression of higher surface irregularity. In contrast to previously published studies that also quantify surface irregularity, we were also able to reveal differences between atypical and anaplastic meningiomas [11, 24]. Similarly to SR, a decrease in sphericity corresponds to less spherical shape of the tumor. Although conceptually different, a decrease in the roundness of a tumor also leads to a decrease in the shape-based features elongation and flatness. This seems counterintuitive at first, but in the case of pyradiomics, these two features are calculated inversely for computational reasons (https://pyradiomics.readthedocs.io/en/latest/features.html). It can therefore be assumed that with increasing tumor proliferation and grade in meningiomas, the tumor surface becomes noticeably more irregular as an expression of a more aggressive growth pattern.

A systematic review published in 2023 [25] found a good AUROC with regard to radiomics-based grading of meningiomas. Though, the vast majority of studies only differentiated between benign and higher-grade meningiomas and only a small proportion of studies investigated all tumor grades separately. Our analysis showed good AUROCs for both sphericity and SR when discriminating between benign and anaplastic meningiomas, but also when distinguishing between benign and atypical versus anaplastic meningiomas. This could be due to the high proliferative activity described in anaplastic meningiomas, which also influences the tumor shape. Interestingly, ROC analysis did not reveal meaningful differential power in the differentiation of benign meningiomas from higher-grade meningiomas. Sphericity alone was acceptable here with an AUROC of roughly more than 0.7. One reason for this could be the histopathological diagnosis criteria for meningiomas. It is conceivable that some benign meningiomas may show CNS infiltration (which would result in an upgrade to an atypical meningioma), but this cannot be proven from the available resection tissue. Furthermore, although CNS infiltration remains a histopathologic criterion for atypical meningiomas [4], its sole use for categorization and prognostic assessment has been questioned [8, 26].

The significant but weak correlation of the mitotic rate with shape-based features and SR suggests that, as an expression of increased proliferative activity, it can also promote a more irregular tumor shape. In atypical meningiomas, for example, the underlying mitotic count can vary substantially according to the WHO classification. The inclusion of additional imaging biomarkers may facilitate risk assessment within a tumor grade. For example, currently investigated histopathologic features such as CNS infiltration or mitotic rate alone do not appear to be suitable for estimating progression-free survival in atypical meningiomas [27]. An MRI-based radiomics study was able to predict an increased rate of mitosis, but the relevance in practice was limited as there were hardly any cases with more than one mitosis/10 microscopic high-power fields and thus no feature showed a relevant correlation with the mitosis rate [28]. However, numerous other factors that are difficult to model, such as the location of the tumor and its relationship to neighboring structures, cause changes in tumor shape.

The incorporation of supplementary MRI sequences into a multiparametric model has the potential to enhance the predictive capacity of radiomics [29]. Similarly, the use of conventional MRI techniques, such as perfusion and diffusion imaging, may already assist in differentiating between benign and higher-grade meningiomas [30].

In addition to the study’s retrospective design, there are further limitations. There is an imbalance between the higher tumor grades due to the relatively rare anaplastic meningiomas. Specifically, the here performed regression analyses may have been biased by inherent heteroscedasticity. Moreover, suggested cut-off values in this discovery cohort have not been validated in an independent validation cohort. Therefore, further multicenter investigation is warranted to establish the reported findings and to include more anaplastic meningiomas. Second, the SR method proposed by Perez [9] and the surface factor utilized by Popadic or Delgado-Lopez [11, 24] seem to be both suitable for the description of the fractal dimension or deviation of the tumor shape in relation to a perfect sphere. Nevertheless, there are differences in the calculation, which means that the numerical values of SR and surface factor are not directly comparable, even though both parameters are functionally dependent [31]. A comparison with a previous study [31] revealed a lower SR across all meningioma grades in our cohort. A comprehensive comparison is not possible due to the lack of reported meningioma grades in the previous study and the lack of comparability of tumor segmentations.

In conclusion, the shape-based radiomics feature sphericity and SR calculated from standard presurgical contrast-enhanced T1w MRI can assist in the preoperative grading of meningiomas and may serve as non-invasive biomarkers.

## Data Availability

The anonymized data presented in the study here are available on reasonable request from the author
